# *catena*-Poly[[(1,10-phenanthroline)cobalt]-μ-2,4′-oxydibenzoato]

**DOI:** 10.1107/S1600536812031534

**Published:** 2012-07-18

**Authors:** Hai-Kang Guo, Feng Fu, Long Tang, Xiang-Yang Hou, Jia Cao

**Affiliations:** a Department of Chemistry and Chemical Engineering, Shaanxi Key Laboratory of Chemical Reaction Engineering, Yan’an University, Yan’an, Shaanxi 716000, People’s Republic of China

## Abstract

In the title compound, [Co(C_14_H_8_O_5_)(C_12_H_8_N_2_)]_*n*_, the Co^II^ atom is six-coordinated in a distorted octa­hedral coordination geometry by four O atoms from two chelating carboxyl­ate groups from different 2,4′-oxydibenzoate anions and by two N atoms from a 1,10-phenanthroline (phen) ligand. The two benzene rings of the 2,4′-oxydibenzoate ligand form a dihedral angle of 77.14 (16)°. Adjacent Co^II^ atoms are bridged by 2,4′-oxydibenzoate anions to form a helical chain that propagates along the *b*-axis direction. Neighboring chains are further assembled by inter­molecular π–π stacking inter­actions between inversion-related phen ligands [centroid-to-centroid distance = 4.0869 (8) Å] to form a two-dimensional supra­molecular architecture.

## Related literature

For related structures and the properties of coordination polymers, see: Han *et al.* (2005 ▶); Xue *et al.* (2009 ▶); Sun *et al.* (2010 ▶); Wang *et al.* (2010 ▶).
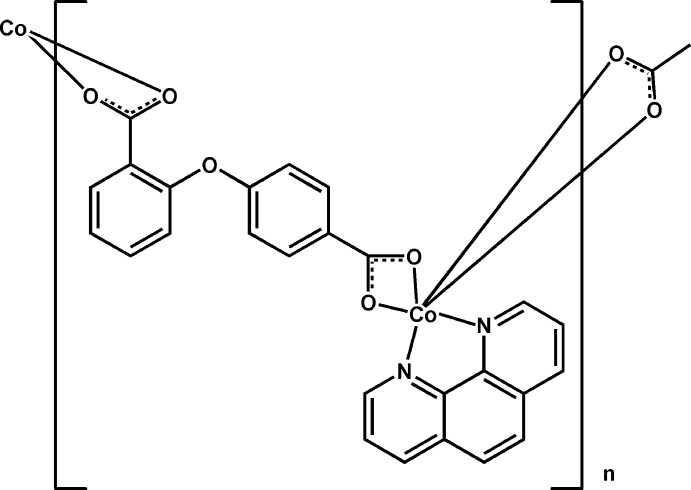


## Experimental

### 

#### Crystal data

[Co(C_14_H_8_O_5_)(C_12_H_8_N_2_)]
*M* *_r_* = 495.34
Monoclinic, 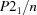

*a* = 7.8524 (16) Å
*b* = 15.345 (3) Å
*c* = 18.778 (4) Å
β = 99.72 (3)°
*V* = 2230.3 (8) Å^3^
*Z* = 4
Mo *K*α radiation
μ = 0.81 mm^−1^
*T* = 293 K
0.40 × 0.20 × 0.15 mm

#### Data collection

Bruker SMART diffractometer
Absorption correction: multi-scan (*SADABS*; Sheldrick, 1996 ▶) *T*_min_ = 0.782, *T*_max_ = 0.898
18945 measured reflections
3921 independent reflections
2794 reflections with *I* > 2σ(*I*)
*R*_int_ = 0.066

#### Refinement

*R*[*F* ^2^ > 2σ(*F* ^2^)] = 0.073
*wR*(*F* ^2^) = 0.141
*S* = 1.15
3921 reflections
307 parameters
H-atom parameters constrained
Δρ_max_ = 0.29 e Å^−3^
Δρ_min_ = −0.35 e Å^−3^

### 

Data collection: *SMART* (Bruker, 1997 ▶); cell refinement: *SAINT* (Bruker, 1997 ▶); data reduction: *SAINT*; program(s) used to solve structure: *SHELXS97* (Sheldrick, 2008 ▶); program(s) used to refine structure: *SHELXL97* (Sheldrick, 2008 ▶); molecular graphics: *DIAMOND* (Brandenburg & Putz, 2005 ▶); software used to prepare material for publication: *SHELXTL* (Sheldrick, 2008 ▶).

## Supplementary Material

Crystal structure: contains datablock(s) I, global. DOI: 10.1107/S1600536812031534/pk2423sup1.cif

Structure factors: contains datablock(s) I. DOI: 10.1107/S1600536812031534/pk2423Isup2.hkl

Additional supplementary materials:  crystallographic information; 3D view; checkCIF report

## Figures and Tables

**Fig. 1.**
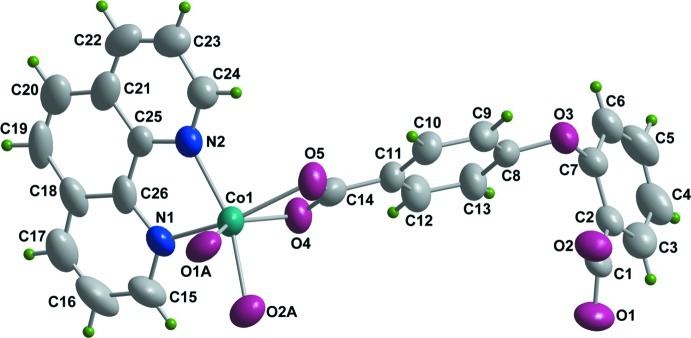
The coordination environment of CoII atoms in the title compound, with thermal ellipsoids drawn at the 50% level. Symmetry code: A -x + 3/2, y + 1/2, -z + 3/2.

**Fig. 2.**
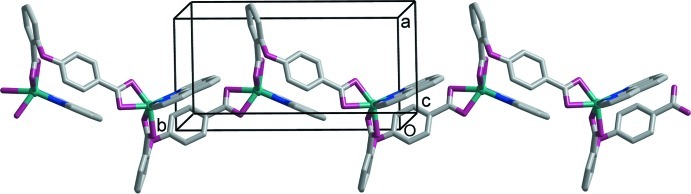
The helical chain formed by molecules of the title compound that extends along b-axis.

**Fig. 3.**
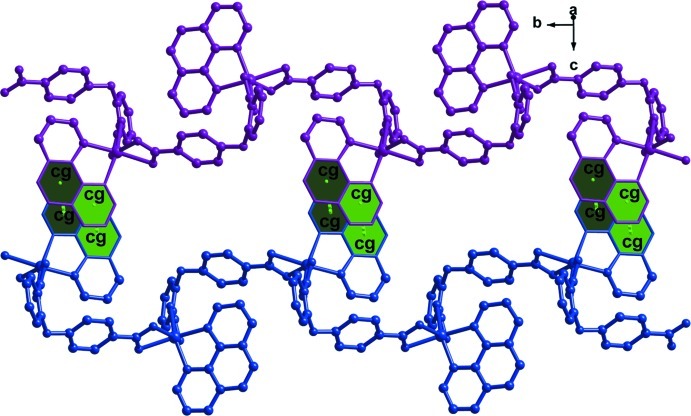
The 2D supramolecular structure formed through π–π interactions.

## supplementary crystallographic information

### Comment

The rational design and syntheses of metal–organic frameworks has been of increasing interest in the crystal engineering of coordination polymers owing to their ability to provide diverse assemblies with fascinating topological structures and material properties (Han *et al.*, 2005; Xue *et al.*,2009). The semi-rigid V-shaped multi-carboxylate ligands with two benzene rings, which contain a central molecular framework that can be bridged by an oxygen atom, have sufficient flexibility that they can freely twist around the oxygen atom, leading to metal complexes with diverse structures in the assembly process (Sun *et al.*, 2010; Wang *et al.*, 2010).

The asymmetric unit contains one Co^II^ ion, one 1,10-phenanthroline ligand and one 2,4'-oxydibenzoate anion. Each Co^II^ atom has a distorted octahedral geometry and is six-coordinated by four O atoms from two chelating carboxylate groups of non-symmetry related 2,4'-oxydibenzoate ligands and by two N atoms from a 1,10-phenanthroline molecule (Fig. 1). The Co—O bond distances vary from 2.077 (3) to 2.201 (3) Å and the Co—N bond lengths are 2.077 (4) and 2.107 (4) Å. Adjacent Co^II^ atoms are linked by 2,4'-oxydibenzoate ligands with carboxyl groups to form infinite one-dimensional helical chains along the b-axis direction (Fig. 2). Neighboring chains are further assembled by intermolecular π–π stacking interaction between the phenanthroline ring systems with a ring centroid-centroid distance of 4.0869 (8) Å, forming a two-dimensional supramolecular architecture (Fig. 3).

### Experimental

A mixture of CoSO_4_.7H_2_O (0.0149, 0.05 mmol), 2,4'-oxybis(benzoic acid) (0.0129, 0.05 mmol), 1,10-phenanthroline (0.0099 g, 0.05 mmol), H_2_O (8 ml) was sealed in 25 ml Teflon-lined stainless steel reactor, which was heated to 413 K for 5 d and was subsequently cooled slowly to room temperature. Red block-shaped crystals were collected in 47% yield based on Co.

### Refinement

All H atoms were positioned geometrically (C—H = 0.93Å) and allowed to ride on their parent atoms, with *U*_iso_(H) values equal to 1.2*U*_eq_(C).

### Crystal data

**Table:**

[Co(C_14_H_8_O_5_)(C_12_H_8_N_2_)]	*F*(000) = 1012
*M**_r_* = 495.34	*D*_x_ = 1.475 Mg m^−^^3^
Monoclinic, *P*2_1_/*n*	Mo *K*α radiation, λ = 0.71073 Å
Hall symbol: -P 2yn	Cell parameters from 5540 reflections
*a* = 7.8524 (16) Å	θ = 3.0–25.4°
*b* = 15.345 (3) Å	µ = 0.81 mm^−^^1^
*c* = 18.778 (4) Å	*T* = 293 K
β = 99.72 (3)°	Block, red
*V* = 2230.3 (8) Å^3^	0.40 × 0.20 × 0.15 mm
*Z* = 4	

### Data collection

**Table:**

Bruker SMART diffractometer	3921 independent reflections
Radiation source: fine-focus sealed tube	2794 reflections with *I* > 2σ(*I*)
Graphite monochromator	*R*_int_ = 0.066
φ and ω scans	θ_max_ = 25.0°, θ_min_ = 3.0°
Absorption correction: multi-scan (*SADABS*; Sheldrick, 1996)	*h* = −9→8
*T*_min_ = 0.782, *T*_max_ = 0.898	*k* = −18→15
18945 measured reflections	*l* = −22→22

### Refinement

**Table:**

Refinement on *F*^2^	Primary atom site location: structure-invariant direct methods
Least-squares matrix: full	Secondary atom site location: difference Fourier map
*R*[*F*^2^ > 2σ(*F*^2^)] = 0.073	Hydrogen site location: inferred from neighbouring sites
*wR*(*F*^2^) = 0.141	H-atom parameters constrained
*S* = 1.15	*w* = 1/[σ^2^(*F*_o_^2^) + (0.0374*P*)^2^ + 2.4235*P*] where *P* = (*F*_o_^2^ + 2*F*_c_^2^)/3
3921 reflections	(Δ/σ)_max_ = 0.003
307 parameters	Δρ_max_ = 0.29 e Å^−^^3^
0 restraints	Δρ_min_ = −0.35 e Å^−^^3^

### Special details

**Table:**

Geometry. All esds (except the esd in the dihedral angle between two l.s. planes) are estimated using the full covariance matrix. The cell esds are taken into account individually in the estimation of esds in distances, angles and torsion angles; correlations between esds in cell parameters are only used when they are defined by crystal symmetry. An approximate (isotropic) treatment of cell esds is used for estimating esds involving l.s. planes.
Refinement. Refinement of F^2^ against ALL reflections. The weighted R-factor wR and goodness of fit S are based on F^2^, conventional R-factors R are based on F, with F set to zero for negative F^2^. The threshold expression of F^2^ > 2sigma(F^2^) is used only for calculating R-factors(gt) etc. and is not relevant to the choice of reflections for refinement. R-factors based on F^2^ are statistically about twice as large as those based on F, and R-factors based on ALL data will be even larger.

### Fractional atomic coordinates and isotropic or equivalent isotropic displacement parameters (Å^2^)

**Table:**

	*x*	*y*	*z*	*U*_iso_*/*U*_eq_	
O4	0.6161 (4)	0.7767 (2)	0.64914 (18)	0.0608 (9)	
O5	0.8475 (5)	0.7208 (2)	0.61748 (19)	0.0631 (9)	
C14	0.6932 (7)	0.7120 (3)	0.6281 (3)	0.0535 (12)	
C11	0.6083 (6)	0.6252 (3)	0.6175 (2)	0.0483 (11)	
C12	0.4465 (7)	0.6116 (3)	0.6354 (3)	0.0630 (14)	
H12	0.3881	0.6578	0.6524	0.076*	
C10	0.6901 (6)	0.5560 (3)	0.5906 (2)	0.0562 (13)	
H10	0.7981	0.5640	0.5776	0.067*	
C13	0.3704 (7)	0.5299 (3)	0.6283 (3)	0.0632 (14)	
H13	0.2624	0.5210	0.6411	0.076*	
Co1	0.66874 (8)	0.35628 (4)	0.84782 (3)	0.0515 (2)	
O2	0.5626 (4)	0.3396 (2)	0.73244 (17)	0.0595 (9)	
C19	0.8855 (7)	0.6524 (3)	0.9794 (4)	0.0756 (18)	
H19	0.9317	0.7083	0.9855	0.091*	
O3	0.3900 (4)	0.3788 (2)	0.59100 (17)	0.0586 (9)	
N2	0.6821 (5)	0.4027 (2)	0.9525 (2)	0.0516 (10)	
C6	0.0948 (7)	0.3431 (3)	0.5734 (3)	0.0661 (14)	
H6	0.0913	0.3523	0.5242	0.079*	
C26	0.7996 (6)	0.5268 (3)	0.9018 (3)	0.0525 (12)	
C1	0.4169 (6)	0.3533 (3)	0.7488 (3)	0.0507 (11)	
O1	0.4041 (4)	0.3737 (2)	0.81312 (18)	0.0678 (10)	
C7	0.2449 (6)	0.3566 (3)	0.6210 (3)	0.0523 (12)	
N1	0.7779 (5)	0.4804 (2)	0.8387 (2)	0.0566 (10)	
C2	0.2544 (6)	0.3446 (3)	0.6948 (2)	0.0476 (11)	
C8	0.4557 (6)	0.4624 (3)	0.6022 (2)	0.0502 (12)	
C18	0.8689 (6)	0.6116 (3)	0.9098 (3)	0.0653 (15)	
C17	0.9161 (7)	0.6477 (4)	0.8483 (4)	0.0824 (19)	
H17	0.9620	0.7037	0.8504	0.099*	
C24	0.6333 (6)	0.3639 (4)	1.0079 (3)	0.0619 (13)	
H24	0.5872	0.3081	1.0010	0.074*	
C4	−0.0473 (7)	0.3059 (4)	0.6720 (4)	0.0783 (18)	
H4	−0.1467	0.2898	0.6894	0.094*	
C9	0.6133 (7)	0.4752 (3)	0.5827 (3)	0.0568 (13)	
H9	0.6690	0.4292	0.5641	0.068*	
C23	0.6459 (7)	0.4005 (4)	1.0763 (3)	0.0736 (16)	
H23	0.6098	0.3698	1.1138	0.088*	
C5	−0.0513 (7)	0.3157 (4)	0.5990 (4)	0.0784 (17)	
H5	−0.1523	0.3039	0.5668	0.094*	
C22	0.7118 (7)	0.4819 (4)	1.0872 (3)	0.0720 (16)	
H22	0.7215	0.5077	1.1325	0.086*	
C20	0.8358 (7)	0.6121 (4)	1.0358 (4)	0.0783 (18)	
H20	0.8483	0.6409	1.0800	0.094*	
C21	0.7651 (6)	0.5271 (4)	1.0304 (3)	0.0627 (15)	
C15	0.8250 (7)	0.5179 (4)	0.7816 (3)	0.0729 (16)	
H15	0.8106	0.4877	0.7380	0.087*	
C16	0.8963 (8)	0.6026 (4)	0.7850 (4)	0.087 (2)	
H16	0.9296	0.6274	0.7442	0.104*	
C3	0.1048 (7)	0.3200 (3)	0.7192 (3)	0.0627 (14)	
H3	0.1069	0.3127	0.7685	0.075*	
C25	0.7487 (6)	0.4845 (3)	0.9629 (3)	0.0509 (12)	

### Atomic displacement parameters (Å^2^)

**Table:**

	*U*^11^	*U*^22^	*U*^33^	*U*^12^	*U*^13^	*U*^23^
O4	0.069 (2)	0.043 (2)	0.072 (2)	−0.0033 (17)	0.0164 (18)	−0.0001 (17)
O5	0.068 (2)	0.046 (2)	0.078 (3)	−0.0039 (18)	0.0199 (19)	0.0048 (17)
C14	0.063 (3)	0.048 (3)	0.048 (3)	0.000 (3)	0.005 (2)	0.010 (2)
C11	0.054 (3)	0.040 (3)	0.051 (3)	−0.008 (2)	0.009 (2)	0.003 (2)
C12	0.065 (3)	0.043 (3)	0.083 (4)	−0.001 (3)	0.018 (3)	−0.004 (3)
C10	0.057 (3)	0.055 (3)	0.058 (3)	−0.008 (3)	0.016 (2)	0.007 (3)
C13	0.066 (3)	0.047 (3)	0.081 (4)	−0.012 (3)	0.025 (3)	−0.007 (3)
Co1	0.0622 (4)	0.0414 (4)	0.0508 (4)	0.0034 (3)	0.0096 (3)	−0.0037 (3)
O2	0.052 (2)	0.071 (2)	0.057 (2)	0.0007 (17)	0.0132 (16)	−0.0115 (17)
C19	0.064 (4)	0.036 (3)	0.111 (5)	0.008 (3)	−0.029 (3)	−0.022 (3)
O3	0.072 (2)	0.050 (2)	0.056 (2)	−0.0153 (17)	0.0190 (17)	−0.0088 (16)
N2	0.058 (2)	0.041 (2)	0.056 (3)	0.0075 (19)	0.011 (2)	0.0021 (19)
C6	0.074 (4)	0.052 (3)	0.068 (4)	−0.010 (3)	0.000 (3)	−0.009 (3)
C26	0.045 (3)	0.036 (3)	0.071 (4)	0.009 (2)	−0.005 (2)	−0.001 (2)
C1	0.064 (3)	0.036 (2)	0.054 (3)	0.001 (2)	0.014 (2)	−0.008 (2)
O1	0.072 (2)	0.084 (3)	0.049 (2)	0.0129 (19)	0.0150 (17)	−0.0154 (19)
C7	0.060 (3)	0.045 (3)	0.053 (3)	−0.009 (2)	0.010 (2)	−0.012 (2)
N1	0.071 (3)	0.044 (2)	0.053 (3)	0.008 (2)	0.005 (2)	0.011 (2)
C2	0.050 (3)	0.042 (3)	0.052 (3)	−0.004 (2)	0.014 (2)	−0.012 (2)
C8	0.069 (3)	0.040 (3)	0.042 (3)	−0.007 (2)	0.009 (2)	0.001 (2)
C18	0.057 (3)	0.043 (3)	0.088 (4)	0.010 (2)	−0.013 (3)	0.015 (3)
C17	0.070 (4)	0.050 (3)	0.114 (6)	−0.004 (3)	−0.025 (4)	0.012 (4)
C24	0.066 (3)	0.060 (3)	0.061 (3)	0.007 (3)	0.013 (3)	0.002 (3)
C4	0.056 (3)	0.073 (4)	0.111 (5)	−0.017 (3)	0.027 (3)	−0.032 (4)
C9	0.072 (3)	0.044 (3)	0.058 (3)	0.001 (3)	0.020 (3)	−0.003 (2)
C23	0.072 (4)	0.089 (5)	0.061 (4)	0.020 (3)	0.013 (3)	−0.002 (3)
C5	0.061 (4)	0.071 (4)	0.097 (5)	−0.003 (3)	−0.005 (3)	−0.031 (4)
C22	0.067 (4)	0.094 (5)	0.053 (4)	0.031 (3)	0.004 (3)	−0.015 (3)
C20	0.074 (4)	0.067 (4)	0.086 (5)	0.022 (3)	−0.011 (3)	−0.017 (4)
C21	0.050 (3)	0.057 (3)	0.074 (4)	0.020 (3)	−0.011 (3)	−0.020 (3)
C15	0.083 (4)	0.073 (4)	0.060 (4)	0.000 (3)	0.003 (3)	0.019 (3)
C16	0.077 (4)	0.080 (4)	0.097 (5)	−0.007 (3)	−0.005 (4)	0.047 (4)
C3	0.061 (3)	0.060 (3)	0.072 (4)	−0.006 (3)	0.025 (3)	−0.010 (3)
C25	0.050 (3)	0.045 (3)	0.054 (3)	0.016 (2)	−0.002 (2)	−0.004 (2)

### Geometric parameters (Å, º)

**Table:**

O4—C14	1.261 (5)	C6—H6	0.9300
O4—Co1^i^	2.077 (3)	C26—N1	1.369 (6)
O5—C14	1.267 (5)	C26—C18	1.408 (7)
O5—Co1^i^	2.190 (3)	C26—C25	1.432 (7)
C14—C11	1.488 (6)	C1—O1	1.269 (5)
C14—Co1^i^	2.473 (5)	C1—C2	1.495 (6)
C11—C10	1.381 (6)	C7—C2	1.387 (6)
C11—C12	1.384 (6)	N1—C15	1.324 (6)
C12—C13	1.385 (6)	C2—C3	1.384 (6)
C12—H12	0.9300	C8—C9	1.363 (6)
C10—C9	1.375 (6)	C18—C17	1.385 (8)
C10—H10	0.9300	C17—C16	1.363 (8)
C13—C8	1.368 (6)	C17—H17	0.9300
C13—H13	0.9300	C24—C23	1.390 (7)
Co1—N2	2.077 (4)	C24—H24	0.9300
Co1—O4^ii^	2.077 (3)	C4—C5	1.374 (8)
Co1—O1	2.087 (3)	C4—C3	1.379 (7)
Co1—N1	2.107 (4)	C4—H4	0.9300
Co1—O5^ii^	2.190 (3)	C9—H9	0.9300
Co1—O2	2.201 (3)	C23—C22	1.354 (8)
Co1—C14^ii^	2.473 (5)	C23—H23	0.9300
O2—C1	1.252 (5)	C5—H5	0.9300
C19—C20	1.342 (8)	C22—C21	1.395 (8)
C19—C18	1.436 (8)	C22—H22	0.9300
C19—H19	0.9300	C20—C21	1.415 (8)
O3—C8	1.385 (5)	C20—H20	0.9300
O3—C7	1.396 (5)	C21—C25	1.412 (7)
N2—C24	1.312 (6)	C15—C16	1.412 (8)
N2—C25	1.361 (6)	C15—H15	0.9300
C6—C7	1.369 (7)	C16—H16	0.9300
C6—C5	1.382 (7)	C3—H3	0.9300
			
C14—O4—Co1^i^	92.3 (3)	O1—C1—C2	118.2 (4)
C14—O5—Co1^i^	87.1 (3)	C1—O1—Co1	91.8 (3)
O4—C14—O5	119.2 (4)	C6—C7—C2	121.8 (5)
O4—C14—C11	121.2 (4)	C6—C7—O3	116.4 (4)
O5—C14—C11	119.5 (4)	C2—C7—O3	121.7 (4)
O4—C14—Co1^i^	57.1 (2)	C15—N1—C26	117.6 (5)
O5—C14—Co1^i^	62.2 (2)	C15—N1—Co1	129.2 (4)
C11—C14—Co1^i^	177.0 (4)	C26—N1—Co1	113.1 (3)
C10—C11—C12	118.4 (4)	C3—C2—C7	117.4 (4)
C10—C11—C14	120.8 (4)	C3—C2—C1	118.4 (4)
C12—C11—C14	120.8 (4)	C7—C2—C1	124.1 (4)
C11—C12—C13	120.8 (5)	C9—C8—C13	120.5 (4)
C11—C12—H12	119.6	C9—C8—O3	115.1 (4)
C13—C12—H12	119.6	C13—C8—O3	124.3 (4)
C9—C10—C11	120.6 (4)	C17—C18—C26	115.8 (6)
C9—C10—H10	119.7	C17—C18—C19	125.9 (6)
C11—C10—H10	119.7	C26—C18—C19	118.3 (6)
C8—C13—C12	119.4 (5)	C16—C17—C18	121.1 (6)
C8—C13—H13	120.3	C16—C17—H17	119.5
C12—C13—H13	120.3	C18—C17—H17	119.5
N2—Co1—O4^ii^	105.35 (14)	N2—C24—C23	124.4 (5)
N2—Co1—O1	98.04 (14)	N2—C24—H24	117.8
O4^ii^—Co1—O1	147.79 (14)	C23—C24—H24	117.8
N2—Co1—N1	79.09 (16)	C5—C4—C3	119.7 (5)
O4^ii^—Co1—N1	101.12 (15)	C5—C4—H4	120.2
O1—Co1—N1	104.84 (15)	C3—C4—H4	120.2
N2—Co1—O5^ii^	92.31 (14)	C8—C9—C10	120.2 (5)
O4^ii^—Co1—O5^ii^	61.42 (13)	C8—C9—H9	119.9
O1—Co1—O5^ii^	96.30 (14)	C10—C9—H9	119.9
N1—Co1—O5^ii^	158.04 (14)	C22—C23—C24	118.6 (6)
N2—Co1—O2	157.20 (13)	C22—C23—H23	120.7
O4^ii^—Co1—O2	97.45 (13)	C24—C23—H23	120.7
O1—Co1—O2	61.06 (12)	C4—C5—C6	120.0 (5)
N1—Co1—O2	96.71 (14)	C4—C5—H5	120.0
O5^ii^—Co1—O2	98.68 (13)	C6—C5—H5	120.0
N2—Co1—C14^ii^	100.58 (15)	C23—C22—C21	120.0 (5)
O4^ii^—Co1—C14^ii^	30.64 (14)	C23—C22—H22	120.0
O1—Co1—C14^ii^	123.66 (16)	C21—C22—H22	120.0
N1—Co1—C14^ii^	130.70 (17)	C19—C20—C21	122.1 (6)
O5^ii^—Co1—C14^ii^	30.78 (13)	C19—C20—H20	119.0
O2—Co1—C14^ii^	99.04 (14)	C21—C20—H20	119.0
C1—O2—Co1	87.1 (3)	C22—C21—C25	117.6 (5)
C20—C19—C18	121.6 (5)	C22—C21—C20	124.6 (6)
C20—C19—H19	119.2	C25—C21—C20	117.8 (6)
C18—C19—H19	119.2	N1—C15—C16	121.8 (6)
C8—O3—C7	118.2 (4)	N1—C15—H15	119.1
C24—N2—C25	117.5 (4)	C16—C15—H15	119.1
C24—N2—Co1	128.4 (4)	C17—C16—C15	119.5 (6)
C25—N2—Co1	114.1 (3)	C17—C16—H16	120.2
C7—C6—C5	119.5 (5)	C15—C16—H16	120.2
C7—C6—H6	120.2	C4—C3—C2	121.5 (5)
C5—C6—H6	120.2	C4—C3—H3	119.3
N1—C26—C18	124.2 (5)	C2—C3—H3	119.3
N1—C26—C25	116.5 (4)	N2—C25—C21	122.0 (5)
C18—C26—C25	119.3 (5)	N2—C25—C26	117.1 (4)
O2—C1—O1	119.8 (4)	C21—C25—C26	120.9 (5)
O2—C1—C2	122.0 (4)		

Symmetry codes: (i) −*x*+3/2, *y*+1/2, −*z*+3/2; (ii) −*x*+3/2, *y*−1/2, −*z*+3/2.
